# A Visit to Washington

**Published:** 1847-03

**Authors:** 


*A Visit to Washington.*—We have long since regarded intercourse with skilful men in the profession, as one of the very best sources of improvement.

The American Society of Dental Surgeons, had its origin in this belief, and we think its founders were by no means mistaken in the estimate in which they held the benefits of interchanging experience by members of the profession.

In a recent visit to the city of Washington, we had an admirable proof of this, in a long interview with our friend Dr. E. Maynard. We anticipated a treat, and were by no means disappointed.

Dr. M. having just returned from Europe, where he has taken the utmost pains to "gather up the fragments" scattered over every part from sunny France to frozen Russia, was enabled to give us a very clear idea of the state of transatlantic dentistry. To us, who have long known his merit, it was particularly gratifying to know that his excellence as an operator, was by no means underrated abroad.

In this country, the dentist considers himself well dealt by, if he receives a fair pecuniary remuneration for his services; but it would appear that peculiar merit is more liberally rewarded in some of the monarchical countries, than in this land of freedom and independence. Dr. M. not only had the offer of the highest titles which the Emperor of Russia could bestow upon a professional man, but a still more substantial exhibition of imperial favor.

We can in no way give a better idea of the manner in which he was received in Russia, than by publishing an extract from one of his letters in answer to some inquiries which we had made in relation to his tour, his success, and the estimate placed upon Dental Surgery by the Russians, and the inducements held out for him to remain there. Upon the last of these topics his reply was as follows:

"The inducements held out to me to remain at St. Petersburg were as follows: by Imperial favor to have the free practice of the city without the usual examinations, licenses, or charges: to be Actual Dentist to His Imperial Majesty and the Imperial family, with the title of "Imperial Dentist," and the rank of Major, equivalent to the 8th or 9th grade of Nobility.

"The magnificent Imperial present to which you allude is a ring, set with sixty diamonds, many of them of large size, around an enormous carbuncle of transcendant beauty. Its *commercial* value I neither know nor care to know: its value *to me* is regulated by the estimate placed by the giver upon the services for which it was given. It was presented to me by that most remarkable personage, Her Imperial Highness, the Grand Duchess Helen, accounted by many, as well for her rare beauty, learning and accomplishments, as for her political importance, 'the first woman in Europe.'

"I deem it proper to say, that should any of our professional brethren think of trying their fortunes in Russia on the strength of my success, I would counsel them to remember that the bare fact of their *going there to practice, without being sent for,* would create a distrust of their abilities that no recommendations or influence could remove. Whatever their skill or reputation at home, time, and much time, would elapse before they would even be consulted by the Imperial family: and even then some trifling circumstance might blast their budding prospects and consign them to professional disgrace.

"If it be objected to this that such was not the case with me—the answer is, that I did not go there to practice: and that my almost immediate and unparalleled success was owing to a train of circumstances, so curiously wrought, so entirely unusual and beyond all human calculation of probabilities, as to furnish one of the strongest arguments against going there upon speculation."

Although we were greatly interested in his description of "Foreign Affairs," yet, in the disposition of our time, we mainly went upon the principle that "charity begins at home," and compared notes upon almost every item of dental *practice.* It is a great pleasure to do this with practical men—those who have carried into actual experiment what too many leave in theory.

On entering his drawing room, our attention was arrested by a written notice posted on the wall. On examining it, we found it read as follows, viz.

"Notice.—Doctor Maynard wishes it to be distinctly understood, and *remembered*, that he has no time to waste in *hearing long stories, being bantered about prices, keeping books*, or *collecting accounts*."

It occurred to us that this was a very appropriate notice, and that we should like to see more walls papered in this way.

Among other items of *practice*, our attention turned upon the use of arsenious acid for destroying nerves in teeth. Our views upon this subject have been heretofore freely expressed, and our reasons for those views.

Some of the objections which we have urged to its use, Dr. M. has, by his peculiar mode of operating, wholly overcome. His method of putting this substance in a tooth, precludes the possibility of its "producing mischievous consequences by its getting into the mouth." We had an opportunity while there, of seeing him operate, where he had the day before placed arsenious acid in a tooth, for the purpose of destroying a nerve, and we will briefly describe the case. His patient was an elderly gentleman, himself one of the oldest and most respectable members of the profession. Dr. M. removed the "packing" in our presence, and exposed the nerve. When his probe first touched it, the patient exclaimed, "'tis not dead yet." Dr. M. observed, that the mere fact that sensation was produced, was not a sure sign the nerve was not dead. His explanation was that the nerve cavity was filled with a semi-fluid substance, and hence, pressure on the lower extremity would be readily felt at the other extremity, and perhaps even *through* the root. By excavating around the natural orifice, and being careful to bring the matter it contained *downwards*, he soon proved that the nerve *was* nearly all dead, by removing the greatest part of it.

His method of confining the arsenic in the cavity is peculiar, and is well calculated to free the nerve from all pressure, to which he attributes most of the pain caused by this operation, and to secure it firmly in its place, excluding even any moisture from the saliva. It is as follows: he takes white wax and works into it cotton or lint, till it is thoroughly mixed together. With this he fills the cavity in the tooth. Before doing this, he exposes the nerve as much as possible, applies the arsenic, and *caps* the orifice with a plate of lead, of a cup shape, the convex side outward. While this is carefully kept in place, he fills the cavity with this cotton and wax, very carefully, and perfectly, in a way so as not to shut in and compress any air which might press upon the nerve. On removing this packing in the case above alluded to, I was surprised to see that his preparation had been kept perfectly dry, although it had been there twenty-four hours.

After he removed this packing, and the preparation, he proceeded to remove the nerve. Instead of attempting to do this at once, he began by cutting on every side of the orifice, so much enlarging it as to be enabled to remove the nerve without pressing the contents of the cavity upwards.

It was to us a matter of interest to examine the instruments used, particularly for removing the nerve.

Some of his probes were made from the mainspring of a watch, by filing or grinding them sufficiently narrow, to enter the smallest space which he wished to probe. In this way he secures the most perfect *spring temper*, a point not easily attained in so fine an instrument as a probe adapted to this purpose. These probes were bearded by cutting them with a sharp knife—the beards pointing backwards.

With different sizes of these probes, and by enlarging the cavity from time to time, he removes the nerve to the extremity of the root.

His operation of filling the root is characterized by the same neatness and dexterity. His instruments are of the most delicate kind, and are adapted to reach to the end of the fang, although the canal may not be entirely straight. In filling these roots he used very heavy gold, we believe from No. 12 to No. 30.

This is cut into strips corresponding to the diameter of the cavity, and is not doubled. The end of one of these strips is laid upon the end of one of these delicate pluggers, and carefully carried up to the upper extremity of the root. If this is effected, the instrument is withdrawn a slight distance, then returned, carrying with it another portion, till the strip is exhausted. In this way the whole root is filled. The main cavity in the crown is filled afterwards in the usual way.

In respect to the success attending Dr. M's operations of this kind, he informs me that it has been almost entire. We saw several of his patients for whom he had operated in this way—in one case where the operation had been performed on several teeth some years before, when the patient was but 11 years old, and the mouth, so far as we could judge, appeared healthy. One thing is very certain, if Dr. M. does not succeed with it no one else need try, for we are satisfied that he has brought it to greater perfection than any one with whose operations we have ever been acquainted. It will be recollected, however, that his patients, of this class, are generally those who take good care of their teeth, and use the means necessary to success.

The mere fact, that he charges from 10 to $30 for performing the operation, is a sufficient guarantee that it will be confined to those who place a great value upon their teeth. This *charge* would be a sufficient proscriptive measure with most operators, and in most locations. Although his method obviates the objection of the acid getting into the mouth, and requires much less of the arsenic than we had supposed necessary, yet, we cannot admit that even *his* method gives us a *guarantee*, that the morbid influence which is sufficient *within* the root, to *destroy* the nerve, will not, in some cases, or even in any case, extend beyond the apex of the root, and create a disturbance there.

It is an old saying that "the proof of the pudding is in the eating it," and if the *result* in his hands, is as favorable as it appears to be, and since this is undoubtedly the most effectual, if not the only means of destroying the nerve readily, short of the instrument itself, we shall by no means, feel like proscribing it *in his hands*; yet we do still regard it, unless confined to operators who exercise the most *scrupulous caution and judgment,* a practice, pernicious in its tendencies. We have no feeling to gratify upon this subject, except to arrive at correct practice, and if the views we have entertained of it, and which we have freely expressed, are not in accordance with pathological principles, we will cheerfully lay them down so far as they can be shown to be wrong.—*Syracuse Ed.*

NOTE.—We had intended noticing, in the present number of the Journal, the peculiar method employed by our friend and former associate in the editorship of this periodical, Dr. Maynard, of treating a decayed tooth, after the pulp-cavity had become exposed, but Dr. Westcott has anticipated so fully, in his remarks above, what we designed saying, that any thing from us upon the subject, is rendered unnecessary. We may, however, be permitted to add, that although somewhat sceptical, with regard to the practicability of preventing a tooth, for any considerable length of time, after the destruction of its pulp with arsenious acid, from exerting a morbid influence upon the surrounding parts, we have long been apprised of the fact, that the peculiar plan of treatment adopted by Dr. M., has proved highly successful.

We took occasion, in a former number of the Journal, to express our views with regard to the use of arsenic as a dental therapeutic agent, and the propriety of filling a tooth after the destruction of its pulp with it; our remarks, however, were not intended to apply to the peculiar methods which Dr. M. adopts in its employment, but to the manner in which it is generally used, and it was upon the result of such of these cases as had fallen under our own observation, that they were based.—*Balt. Ed.*

